# Perineal resuturing versus expectant management following vaginal delivery complicated by a dehisced wound (PREVIEW): a pilot and feasibility randomised controlled trial

**DOI:** 10.1136/bmjopen-2016-012766

**Published:** 2017-02-10

**Authors:** L Dudley, C Kettle, P W Thomas, K M K Ismail

**Affiliations:** 1 The Maternity Centre, Royal Stoke, University Hospitals of North Midlands, Staffordshire, UK; 2 Faculty of Health, Staffordshire University, Stafford, UK; 3 Faculty of Health Sciences, Professor Emerita of Women's Health, Staffordshire University, Stafford, UK; 4 Faculty of Health and Social Sciences, Professor of Health Care Statistics and Epidemiology, Bournemouth University Clinical Research Unit, Bournemouth University, Dorset, UK; 5 Institute of Metabolism and Systems Research, College of Medical and Dental Sciences, University of Birmingham, Birmingham, UK

**Keywords:** Postnatal, Perineum, Dehiscence, Re-suturing, Expectancy

## Abstract

**Objective:**

To establish the feasibility of conducting a definitive randomised controlled trial (RCT) comparing the effectiveness of resuturing versus expectant management for dehisced perineal wounds.

**Design:**

A multicentre pilot and feasibility RCT.

**Setting:**

Ten UK maternity units from July 2011 to July 2013.

**Population:**

Eligible women with a dehisced perineal wound within 2 weeks of childbirth.

**Methods:**

The interventions were resuturing or expectancy. Randomisation was via web or telephone, stratified by participating centre. Blinding was not possible due to the nature of the interventions. Analysis was by intention-to-treat.

**Outcome:**

The primary outcome measure was wound healing at 6–8 weeks.

**Results:**

The study revealed a number of feasibility issues, particularly strong patient and clinician preference for treatment options at recruiting centres and the timing of the primary outcome measure. Thirty-four women were randomised (17 in each arm). Data from 33 women were analysed on an intention-to-treat analysis to obtain preliminary estimates of effect size. There was a difference in wound healing at 2 weeks favouring resuturing (OR 20.00, 95% CI 2.04 to 196.37, p=0.004). However, by 6–8 weeks all but one wound in both groups had healed.

**Conclusions:**

PREVIEW revealed a number of feasibility issues, which impacted on recruitment rate. These will have to be taken into account in the design of any future definitive study. In this feasibility study, resuturing was associated with quicker wound healing and women reported higher satisfaction rates with the outcome at 3 months.

**Trial registration number:**

ISRCTN05754020.

**Strengths and limitations of this study**

- PREVIEW is the only pilot and feasibility randomised controlled trial to date, comparing resuturing versus expectancy for the management of dehisced perineal wounds following childbirth.
- There are several strengths to PREVIEW including the design, randomisation strategy and standardised management protocol. All women randomised into the study are accounted for, with overall complete follow-up rates of 30 out of 33 women (91%) for the primary outcome measure.
- A weakness of this study was the recruitment rate making estimates of recruitment rate, attrition rate and effect size less precise than intended. Nevertheless, it is important to stress that a fully powered test of clinical effectiveness was not the intention of PREVIEW and our preliminary results will feed into deliberations regarding a plausible effect sizes to inform future sample size calculations.
- Blinding of the interventions was not possible due to the nature of the treatment options.

## Introduction

Perineal trauma affects a vast number of women worldwide, with more than 350 000 women requiring suturing for a spontaneous tear or episiotomy in the UK per year.^1^ Postpartum management of perineal trauma including the prevention of wound infection and assessing wound healing are considered core components of routine maternity care.^2^ ^3^ There is wide variation in reported rates of perineal trauma wound infections. Some reports provide rates of 0.3–10% in perineal trauma wounds in general,^4–6^ while others quote infection rates in association with perineal wound dehiscence, as high as 39–79%.^7–9^ Moreover, identifying the actual prevalence of perineal wound dehiscence has been equally challenging with reported rates ranging from 0.4%^10^ to 13.5%^11^ being published.

Perineal wound dehiscence is a cause of major physical, psychological and social problems. An infected perineal wound is a potential route for systemic infection whereby sepsis and septic shock may ensue.^12^ In England and Wales, between 2006 and 2008, sepsis was identified as the leading cause of maternal mortality.^13^ During this triennium, one of the seven women who died from sepsis after a vaginal delivery had an infected perineum following with a second-degree tear. While subsequent confidential enquiries have demonstrated a reduction in the rates of deaths from sepsis, it remains one of the leading direct causes of mortality in women following vaginal delivery.^14^ ^15^ Morbidity associated with perineal wound dehiscence poses a serious threat to the woman's general well-being and quality of life due to persistent pain and discomfort at the perineal wound site, urinary retention, defaecation problems, dyspareunia, psychological and psychosexual issues from embarrassment and altered body image.^3^ ^10^ This can have a negative impact on the mother's ability to feed and interact with her newborn baby.^16^ Furthermore, this problem impacts on healthcare resources, as some women may require interventions to manage shorter and longer term consequences.^17^

Perineal wound dehiscence is traditionally managed expectantly. However, it can take several weeks for the wound to fully heal. Several retrospective studies^7–9^ ^18^ and two small randomised controlled trials (RCTs)^19^ ^20^ have suggested that secondary perineal repair is a possible alternative management option even in the presence of infection. However, methodological inadequacies of the two studies included in a recent Cochrane review led the authors to conclude that there was an urgent need for a comprehensive clinical trial to identify the best management strategy for dehisced perineal wounds following primary repair of the initial trauma.^21^

## Aims and objectives

The overall aims of this study from the outset were to assess the feasibility of conducting a definitive RCT comparing the effectiveness of resuturing versus expectant management for dehisced perineal wounds and pilot the processes of such a trial. The specific objectives were: to assess the feasibility of the study protocol; gauge participants' acceptability of the research plan; test recruitment and attrition rates and obtain preliminary estimates of effect size to facilitate sample size calculations for the definitive study.

## Methods

### Study design

**Figure 1:**
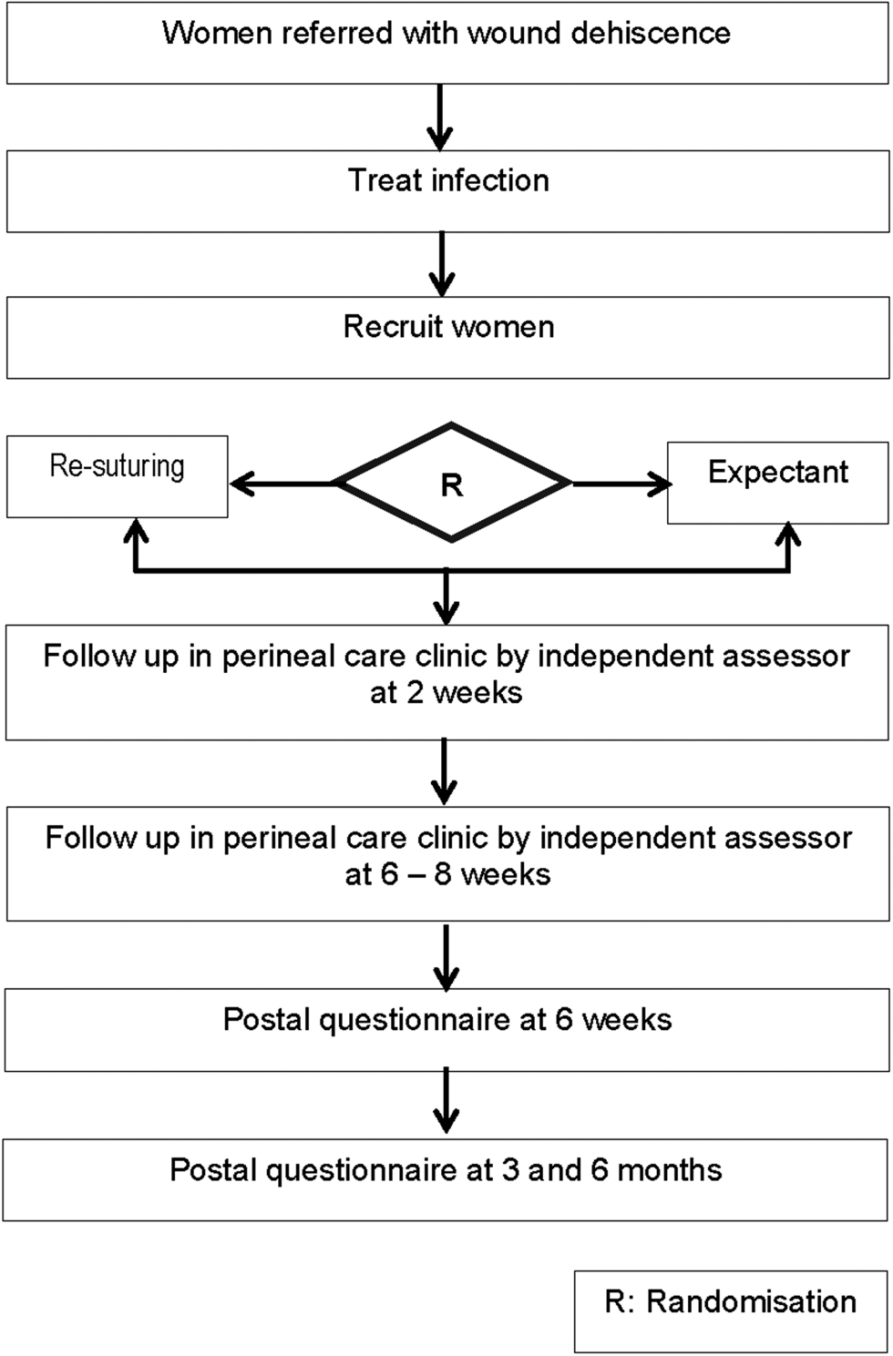
Plan of pilot RCT. RCT, randomised controlled trial.

The study design is briefly described below (figure 1); a more comprehensive study protocol is published elsewhere.^22^

### Study population

Women, with a dehisced perineal wound within the first 2 weeks following a primary repair of a second-degree tear or episiotomy in any of the recruiting sites, were potentially eligible for the RCT.

For the purpose of the study, wound dehiscence was defined as separation of the skin and muscle layers.

Women were excluded from the study if they suffered a pregnancy loss, were <16 years old, were considered to have a high anaesthetic risk, had sustained a perineal trauma higher than a second-degree tear or did not provide a valid written consent to participate.

### Setting

The RCT was conducted in 10 maternity centres in the UK in order to assess realistic recruitment rates and acceptability across different types of units.

### Interventions

Secondary resuturing was compared with expectancy and respective standard operating procedures (SOPs) were developed *(not submitted but available from the trial team).* The SOP for secondary resuturing specified that the procedure was to be conducted in theatre. Regional anaesthesia was recommended, with general anaesthesia for those women who had a contraindication for a regional block.

The recommended suture material for resuturing and the repair techniques for the different dehisced layers are detailed in table 1. The use and type of antibiotics in either arm was left to clinician's discretion.

**Table 1:** Recommended suturing methods and material for the repair of dehisced perineal wounds

Methods	Standard surgical procedures for secondary suturing should be followed including wound debridement if needed
Repair of the vaginal mucosa	Continuous suturing technique
Repair of the perineal muscle	Interrupted sutures
Repair of the skin	Depending on the length of the wound, the skin could be sutured by interrupted or subcutaneous sutures or left unsutured if the edges are approximated by suturing the underlying tissues
Recommended suture material	To ensure standardisation of materials, the PREVIEW study team recommended standard synthetic polyglactin 910 (gauge 2/0) suture material as the material of choice

### Outcome measures for the definitive trial

The primary outcome we proposed for the definitive trial was the proportion of women with a healed wound, at 6–8 weeks following randomisation. Wound healing was defined as no areas of dehiscence observed by a clinician independent from the trial team.

Secondary outcomes suggested included pain, dyspareunia, women's satisfaction with the aesthetic results of the perineal wound and breastfeeding rates.

### Data collection and input

Standardised questionnaires were based on those used and tested by members of the research team in other childbirth-related perineal trauma studies.^23^ ^24^ In addition, a bespoke operating record data collection sheet was developed to enable the assessment of compliance with protocol recommendations. For the primary outcome of wound healing, participants had an assessment of their perineal wound at 2 weeks and 6–8 weeks following randomisation. While for the secondary outcomes, participants were asked to complete a postal questionnaire at 6 weeks, 3 months and 6 months following trial entry. Perineal pain was also assessed by the participants at 2 weeks.

### Sample size

At the initial stage of designing this pilot feasibility trial, sample size considerations were based on precision of estimates of recruitment rate, attrition rate, proportion with healed wound (primary outcome) and preliminary estimate of effect size. A full discussion of sample size considerations for the PREVIEW study was previously published.^22^ However, we unexpectedly encountered strong patients' and clinicians' preferences for the type of management. As a consequence, the actual numbers recruited fell considerably below the initial anticipated sample size of 144 women, despite a formal extension of recruitment period and increasing the number of recruiting centres. Therefore, following discussions with the trial data monitoring committee, results from a reduced sample size of 40 women were deemed adequate to address the main aims of this feasibility study. Rather than the 80% recruitment rate originally assumed, experience in the first part of the trial suggested the true figure was likely to be nearer 25%, which with 40 recruited women would be estimated with a precision (defined as twice the SE) of ±7%. An attrition rate of 20% (producing outcome data on 32 women) would be estimated with a precision of ±12%, healing at 6–8 weeks (assumed to be around 50%) measured on 16 women with outcome data in each arm of the study would be estimated with a precision of around ±25% (±17% for both arms combined).

### Consent and randomisation

All study participants provided valid written informed consent. Women who did not wish to participate in the study were managed in accordance with their local hospital practice. Web-based/telephone-based randomisation was used to allocate study participants. The randomisation schedule was developed by Bristol Randomised Trials Collaboration. The allocation ratio was 1:1 and randomisation was in blocks, stratified by centre.

### Blinding

Owing to the nature of the interventions, it was not feasible to blind outcome assessors, care providers or participants themselves. However, the follow-up perineal wound assessments were undertaken by independent practitioners whenever possible.

### Statistical methods of analysis

Recruitment rate, attrition rate and proportion of women with healed perineal wounds at different time points were estimated (with 95% CIs). Analysis of effect size was undertaken on an intention-to-treat basis, to limit the possibility of bias associated with women not receiving the allocated intervention. Reminder letters and phone calls were used to ensure data on the outcome measures were as complete as possible. No imputation methods were used in the case of missing data.

Statistical analysis was conducted using IBM SPSS 2012 software, V.21, except where otherwise indicated. Baseline characteristics of the comparative groups were summarised using standard descriptive statistics only. Owing to the much smaller than anticipated sample size, spread across a larger number of sites, a simplified version of the analysis described in the published protocol was conducted. For dichotomous data, such as the primary outcome of healed wound at 6–8 weeks, effect size was estimated using ORs (95% CI) calculated using RevMan 5.2 (Review Manager (RevMan) (Computer program). Version 5.2. Copenhagen: The Nordic Cochrane Centre, *The Cochrane Collaboration*, 2012.) and statistical significance tested using Fisher's exact test. No a priori adjustment of baseline covariates, no subgroup analyses and no interim analyses were planned or performed.

## Results

### Recruitment and participant flow

**Figure 2:**
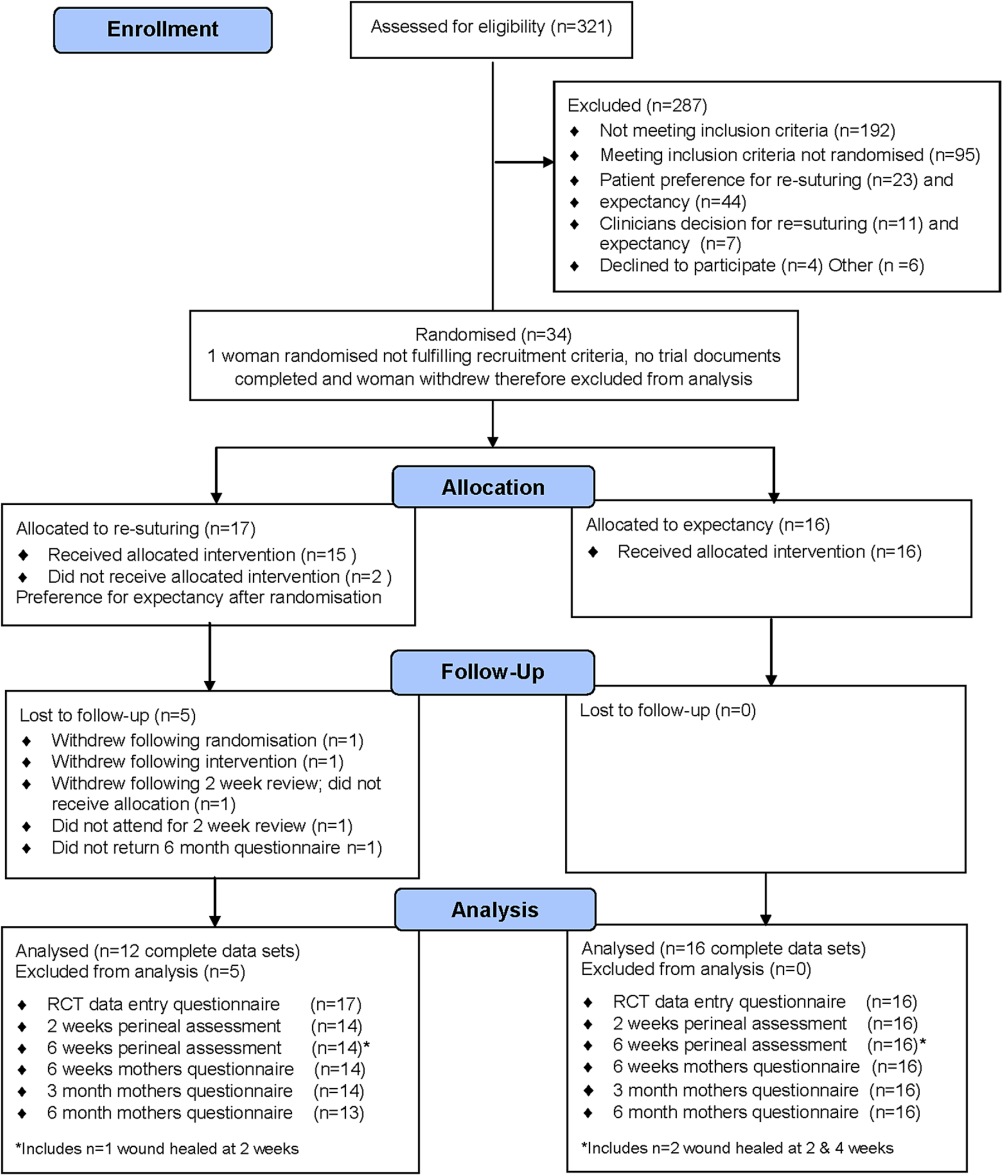
CONSORT flow. CONSORT, Consolidated Standards of Reporting Trials.

The Consolidated Standards of Reporting Trials (CONSORT) flow diagram (figure 2) outlines the progress through the RCT.

Four sites started recruitment on 25 July 2011 with the remaining six sites starting at later dates. One site withdrew from the study in February 2013 due to a lack of a full research team to deliver the study. Recruitment ended on 25 July 2013 and the last participant 6-month questionnaire was returned at the end of January 2014.

### Recruitment and attrition rate

During the recruitment period, members of the PREVIEW team assessed a total of 321 women for eligibility, of these 128 met the trial inclusion criteria. However, only 33 women were randomised into the study (ie, 10% (95% CI 7% to 14%) of the total number screened and 26% (95% CI 19% to 34%) of eligible women) (figure 2).

A total number of 34 women were randomised; however, one of the randomised women did not fulfil recruitment criteria (skin dehiscence only), no trial documents were completed and the woman withdrew, this participant was therefore excluded from the analysis. Seventeen participants were allocated resuturing and 15 received their allocated intervention (two participants expressed a preference for expectancy following randomisation). Sixteen women were randomised to expectant management and all participants received their allocated intervention. All participants were included in the statistical data analysis unless they were lost to follow-up (n=5). Complete data on wound healing outcome were available in 91% (95% CI 76% to 98%) of all participants (14 out of 17 and 16 out of 16 participants in the resuturing and expectancy groups, respectively).

The main reasons for non-randomisation of eligible participants were patient preference to a particular management in 74% (n=70 out of 95) of the cases and clinicians preference for a particular management in 19% (n=18 out of 95) of the cases. Additional reasons were women's decision not to take part 4% (n=4 out of 95) and women not referred to the research team 3% (n=3 out of 95).

*Baseline data*: Baseline antepartum and intrapartum clinical characteristics for each group are provided in table 2. In accordance with CONSORT guidance,^25^ these are reported using standard descriptive statistics.

**Table 2:** Baseline antepartum and intrapartum characteristics at trial entry

	Resutured (n=17) n (%)	Expectancy (n=16) n (%)
Age (years)		
20–24	7 (41)	3 (19)
25–29	3 (18)	5 (31)
30–34	5 (29)	7 (44)
35 and over	2 (12)	1 (6)
Ethnicity		
White	17 (100)	10 (63)
Non-white	0 (0)	6 (37)
BMI (NICE reference range, kg/m^2^)		
Underweight: <18.5	0 (0)	1 (6)
Healthy: 18.5–24.9	5 (29)	10 (63)
Overweight: 25–29.9	7 (42)	2 (12)
Obese: ≥30	5 (29)	3 (19)
Predelivery medical conditions*		
Yes	6 (35)	6 (37)
No	11 (65)	10 (63)
Smoking (woman's self-reported status)		
Yes	3 (18)	1 (6)
No	14 (82)	15 (94)
First vaginal delivery		
Yes	14 (82)	13 (81)
No	3 (18)	3 (19)
Previous perineal trauma		
Yes	3 (18)	3 (19)
No	14 (82)	13 (81)
Previous perineal wound dehiscence (in women with previous perineal trauma)		
Yes	2 (67)	0 (0)
No	1 (33)	3 (100)
*Analgesia used in labour*		
Entonox		
Yes	14 (82)	13 (81)
No	3 (18)	3 (19)
Epidural		
Yes	11 (65)	6 (37)
No	6 (35)	10 (63)
2nd stage of labour in minutes, mean (SD)	80 (64)	93 (66)
Mode of vaginal delivery		
Spontaneous	7 (41)	9 (56)
Operative	10 (59)	7 (44)
Birth weight ≥4 kg		
Yes	3 (18)	2 (12)
No	14 (82)	14 (88)
Meconium liquor present		
Yes	4 (23)	2 (12)
No	11 (65)	14 (88)
Type of perineal trauma		
Information not available	2 (12)	0 (0)
Spontaneous (2nd degree)	5 (29)	4 (25)
Episiotomy	12 (71)	12 (75)
Clinician performing primary repair		
Midwife	7 (41)	8 (50)
Doctor	10 (59)	8 (50)
Vicryl rapide used for repair of 2nd degree tear or episiotomy		
Yes	15 (88)	15 (94)
No	1 (6)	1 (6)
Information not available	1 (6)	0 (0)
Location of perineal repair		
Delivery room	14 (82)	13 (81)
Theatre	3 (18)	3 (19)
Estimated blood loss >500 mLs		
Yes	5 (29)	4 (25)
No	12 (71)	11 (69)
Information not available	0 (0)	1 (6)
Most recent haemoglobin (Hb) <11.0 g/dL		
Yes	5 (29)	4 (25)
No	10 (59)	11 (69)
Information not available	2 (12)	1 (6)
Antibiotics in labour		
Yes	2 (12)	2 (12)
No	15 (88)	14 (88)

*Predelivery medical conditions: resuturing, scoliosis; raised blood pressure; antibiotics for pyelonephritis 1 week prior to birth; bicuspid aortic valve and supra ventricular tachycardia; mild thoracolumbar scoliosis—reported back pain during pregnancy; previous laparoscopy and salpingectomy. Expectancy, factor 5 leiden; possible obstetric cholestasis; mild thrombocytopenia in pregnancy; gestational hypertension on labetalol; hypothyroidism; palpitations and shortness of breath.

BMI, Body Mass Index; NICE, National Institute for Health and Care Excellence.

### Potential clinical outcome measures

We proposed the proportion of wound healing at 6–8 weeks as the primary outcome for the definitive trial. All but one woman (97% (95% CI 83% to 100%)) had healed wounds at that time point (table 3).

**Table 3:** Wound healing

	Resutured n=17 n (%)	Expectancy n=16 n (%)	OR for healing in resutured group (95% CI)	p Value*
2 weeks: postrandomisation			20.00 (2.04 to 196.37)	0.004
Yes	8 (57)	1 (6)		
No	6 (43)	15 (94)		
6 weeks: postrandomisation			0.27 (0.01 to 7.25)†	0.47
Yes	13 (93)	16 (100)		
No	1 (7)	0 (0)		

2 weeks resuturing: Three women not included in analysis as one woman did not attend for review and two women had withdrawn.

6 weeks resuturing: This includes one woman whose wound had healed at 2 weeks, no appointment needed at 6 weeks; three women withdrew and not included in analysis.

6 weeks expectancy: This includes one woman whose wound had healed at 2 weeks and one woman whose wound had healed at 4 weeks.

*p Value, Fishers exact test.

†For the calculation of OR when one of the cells has a value of 0, the software adds a value of 0.5 to all cell counts.

One of the women randomised to resuturing group (received the allocation) had two superficial areas of skin dehiscence at 6 weeks but this went on to complete wound healing by 13 weeks following randomisation. Wound healing was also assessed at 2 weeks and a preliminary estimate of effect size calculated. A significant difference was noted between both groups in favour of resuturing (8 out of 14 (57%) versus 1 out of 16 (6%) OR 20.00, 95% CI 2.04 to 196.37, p=0.004).

We tested the feasibility of collecting several potential secondary outcomes (the results are submitted as online supplementary 1). These include significantly greater satisfaction with healing at 3 months in the resutured group (14 out of 14 felt perineum had healed compared with 11 out of 16 in the expectancy group, p=0.045).

10.1136/bmjopen-2016-012766.supp1 Supplementary data

### Outcomes relevant to the design of any future full trial

#### Protocol adherence

Protocol adherence was good, with only one woman randomised against trial guidance. Nevertheless, while the research plan was acceptable within the recruiting organisations, there are several important issues that should be considered in any future trial to mitigate the risk of bias. These include: the administration of antibiotics, suture materials, repair technique and the independent assessment of wound healing.

#### Administration of antibiotics

Owing to lack of current evidence, antibiotic administration and its type was left to clinicians' discretion. However, we collected data to assess the feasibility of standardising antibiotic regimens in a future study (submitted as online supplementary 2: types of antibiotics prescribed). Out of 33 RCT data entry questionnaires, 79% (n=26) of women had been prescribed antibiotics either prior to or at the point of randomisation in the absence of positive microbiology. For those women who were randomised to resuturing, oral antibiotics were prescribed in 71% (n=12) of women at or before randomisation, intravenous antibiotics were received by 65% (n=11) at the time of resuturing and oral and intravenous antibiotics (at the operative procedure) were received by 53% (n=9) of women (information regarding the administration of intravenous antibiotics at operative procedure was not available for one of the women). For those women randomised to expectancy, oral antibiotics were prescribed in 88% (n=14) of women at or before randomisation (one woman (6%) also received one dose of intravenous antibiotics).

10.1136/bmjopen-2016-012766.supp2 Supplementary data

Microbiology of wound swabs taken at the initial wound assessment revealed that seven of the 26 (27%) women prescribed antibiotics showed no evidence of infection while one out of the seven women not prescribed antibiotics had a positive microbiology result (heavy growth of anaerobic organisms) (14%).

#### Resuturing protocol

There were no protocol violations with regards to timing of resuturing. Despite some organisational barriers, all procedures were conducted in maternity theatres by a senior obstetric registrar or consultant.

Data from the operative records demonstrated that the suture materials detailed in table 1 were used in 60% (9 out of 15) of the secondary repairs (fast absorbing polyglactin was used for skin closure in five women and one record did not detail the type of suture material used).

The vaginal mucosa was intact in eight out of 15 of the secondary repairs. When resutured, compliance with the recommended methods detailed in table 1 was 71.4% (5 out of 7). One record detailed an interrupted method for the vaginal mucosa and one record did not document the method used. Compliance with recommended methods for the perineal muscle layer was 86.6% (13 out of 15). One record detailed an interrupted method for the perineal muscle layer and one record did not document the method used. The skin layer was sutured using the continuous technique in six out of 15 cases, and the interrupted technique in seven out of 15 cases. Two records did not document the method used.

#### Independent assessment of wound healing

The protocol for the RCT recommended that an independent assessor conducted perineal assessments with the intention to limit the introduction of detection bias. Owing to organisational constraints, this was only achieved in 43% (n=13 out of 30) of women at 2 weeks and in 50% (n=14 out of 28) at 6–8 weeks (includes one wound assessed at 4 weeks).

### Women's views

In a nested qualitative study,^26^ women were interviewed as part of the PREVIEW study to explore their physical and psychological experiences following perineal wound dehiscence, to assess the acceptability of the research plan and ensure that all outcomes relevant to women are included in the definitive trial.

### Adverse incidents

There were no adverse incidents reported in either group at any of the recruiting sites.

## Discussion

PREVIEW demonstrated significant differences relating to the rate of wound healing and women's satisfaction with the outcomes at 3 months in favour of resuturing dehisced second-degree tear or episiotomy wounds. However, it is important to emphasise that the main aim of this study from the outset was to assess the feasibility of conducting a full-scale definitive RCT and to pilot the study procedures. We acknowledge that any effect size estimate derived from this study must be interpreted cautiously, and may be imprecise because of the small sample size. Nevertheless, the study provided the most robust clinical evidence to date that should inform any future definitive trials addressing a similar research question with regards to the following issues.

### Feasibility issues for any future trial

Prior to starting the study, we expected some health service delivery issues in relation to resuturing. These included: location for secondary resuturing (delivery suite or gynaecology theatres), categorisation of the procedure (emergency or elective), engagement with the anaesthetic team and arrangements for baby care while women were in the hospital for the procedure. We also aimed to include units with dedicated perineal clinics to facilitate recruitment. However, following active recruitment, the study still faced several organisational barriers including wide variations in referral pathway to their perineal care service, frequency of clinics and service provision outside normal clinic hours. It is for the above reasons that we strongly recommended involving local hospital managers, clinical leads and anaesthetists prior to trial set up and ensure the availability of clear integrated referral pathways between primary and secondary care with regards to postnatal perineal wound problems.

We were only able to recruit 26% of the potentially eligible women despite adopting numerous multifaceted strategies in line with other studies.^27–30^ The main reasons for non-randomisation were secondary to patients' and clinicians' preferences. Our Cochrane review^21^ on the management of dehisced perineal wounds demonstrated the degree of uncertainty with regards to treatment options for dehisced perineal wounds. This confirms that currently, the management of this complication is very much based on custom and tradition rather than evidence. It is plausible that long-standing traditions and previous experience may have resulted in a loss of this uncertainty among some clinicians and hence the equipoise which drives recruitment into clinical trials. Although only speculative, it is possible that this was a potential barrier to clinicians' engagement and subsequent failure to recruit eligible women. Indeed, this has been acknowledged by Preston *et al*^31^ who suggest that healthcare professionals can intentionally or unintentionally act as ‘gatekeepers’, the consequences of which may potentially introduce bias to patient selection, or affect the rate of patient identification and therefore recruitment. It is important to consider this issue in any future trial to ensure that the cohort of multiprofessional clinicians involved in delivering care for women with such complications are in actual equipoise.

We made concerted efforts, at each participating centre, to ensure the availability of a member of the PREVIEW trial team to randomise participants at the point of review of their dehisced wound. Reducing the time a woman spent at the hospital and avoiding a significant increase in workload for staff were crucial towards enhancing recruitment opportunities. However, at times this proved to be challenging. Many National Health Service (NHS) organisations have been undergoing a management of change over recent years and clinicians and researchers in post during study set up and site initiation visits in some sites were moved to other areas or left to take up alternative positions of responsibilities. In addition, as women sometimes presented for review out of normal working hours, there were, potentially, missed recruitment opportunities if a researcher or clinician, who had completed his/her good clinical practice (GCP) training, was not available to consent the woman. This is a particular issue in obstetrics and acute care environments such as accident and emergency and was the focus of a paper by Kenyon *et al*^32^ leading to the development of a standardised tool kit for training clinic staff in GCP activities. In relation to the PREVIEW RCT, it is unrealistic and, indeed, was not favoured by the trial team to expect women at such an early postnatal period to return to the hospital just for the purpose of recruitment into the study.

It is not unexpected that studies, such as PREVIEW, with quite distinct treatment options may face additional challenges with recruitment.^29^ ^33–36^ Women and clinicians in PREVIEW expressed strong preferences for either resuturing or expectancy of the dehisced perineal wound and one solution towards addressing women's preferences would be to include a patient preference trial alongside the traditional RCT conducted when women are in equipoise, thus resulting in a ‘four-armed’ trial as suggested by some authors.^37^ Nevertheless, critics of this approach have argued that comparing non-randomised groups is unreliable particularly if confounding variables are not controlled for and that preferences may change during the trial period.^38–40^ Moreover, there is the potential for unbalanced arms of the trial, as Tincello and associates experienced in their study,^41^ and this would need to be factored into any discussions and sample size calculations. It has also been suggested in some surgical trials that if there is lack of equipoise, where clinicians have preference for particular management techniques, alternative methods of randomisation could be considered where women would be randomised to a treating clinician.^29^ Indeed, the above alternative or additional methods of recruitment should be carefully considered when designing a future definitive trial to mitigate the risk of failure to recruitment. Nevertheless, it is also possible that following the dissemination of information of this study and the potential benefit of resuturing compared with expectant management for dehisced perineal wounds that more stakeholders would, at least, be in equipoise to evaluate either treatment intervention.

PREVIEW had prespecified outcome variables and a control group, although not essential requirements in a pilot study. We acknowledge the on-going debate surrounding hypothesis testing and prespecified outcome variables for pilot and feasibility research. However, a review of pilot and feasibility studies reported that the majority of studies included control groups and conducted and reported hypothesis testing for one or more of the outcome variables.^42^ In PREVIEW, we opted to present preliminary estimates of effect size that will help to inform the design of the future trial.

**Figure 3:**
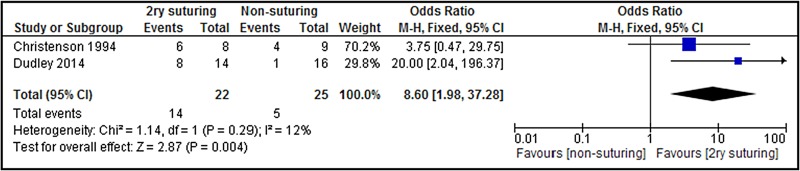
Meta-analysis of two studies for wound healing.

In general, PREVIEW revealed that the prespecified primary outcome measured was feasible to collect. However, we recommend that for a full-scale study careful consideration should be given to revising the timings of assessing this outcome because 6–8 weeks is probably a long duration for women to wait for a wound to heal and the majority of wounds would have healed by that time. Indeed, other studies that have reported on wound healing following secondary resuturing have evaluated this outcome at 2–3 weeks.^8^ ^9^ Similarly, a small RCT of infected episiotomies evaluated wound healing at <4 weeks and found that women who were not resutured experienced longer healing times (>4 weeks) n=4 out of 9 (44%) than women who were resutured 6 out of 8 (75%).^19^ To facilitate a sample size calculation for a full-scale trial, we have pooled our results with those of Christensen^19^ (figure 3).

### Piloting trial processes

The study protocol provided a standardised operating procedure for wound resuturing. While the authors acknowledge that this could be considered a potential disincentive towards clinicians' recruitment, the trial team felt it was important to standardise the management across participating centres. The recommended protocol was adhered to in most of the cases with minor exceptions. We recommend that additional time is dedicated to the provision of resuturing training in any future definitive trial to ensure standardisation of the intervention. The use of antibiotics in PREVIEW was left to the clinician's discretion. Over half of the women who were allocated resuturing also received an additional stat dose of intravenous antibiotics. This ‘cointervention’ could be viewed as a possible source of performance bias. Nevertheless, this was designed as a pragmatic study and the variation in antibiotics use is a true reflection of what actually happens in clinical practice. Collaborative discussions with obstetricians, microbiologists and tissue viability teams need to consider how to avoid or limit this, potential, threat to internal validity in any future study.

The PREVIEW protocol recommended that perineal wound assessments at all-time points were conducted by a clinician independent from the study with the intention to limit the introduction of detection bias. In reality, this was achieved in less than half of all assessments (44%), suggesting that further consideration needs to be given towards achieving independent assessments when planning for the definitive study. Organisational constraints because of service demands and lack of capacity were the main reasons for not achieving higher compliance rates. It has been suggested that where independent assessment is not achievable, two or more individuals assess the outcomes and resolve any disagreements by consensus.^38^ In reality, this would need concerted efforts by committed recruiting sites to achieve in practice.

### Strengths and limitations of the study

There are several strengths to PREVIEW including the design, randomisation strategy and standardised management protocol. All women randomised into the study are accounted for, with overall complete follow-up rates of 30 out of 33 women (91%) for the primary outcome measure. While these follow-up rates are slightly lower than those previously reported in studies investigating secondary perineal repair (94% and 100%, respectively),^19^ ^20^ they are comparable to other obstetric studies which have compared suturing or no suturing for primary perineal repair.^43^ ^44^ It is worth considering incentive strategies^30^ and exploring the potential to follow-up women at their primary care centre to assess wound healing to minimise attrition rates in a future trial. However, the biggest limitation to PREVIEW was the recruitment rate making estimates of recruitment rate, attrition rate and effect size less precise than intended. Nevertheless, it is important to stress that a fully powered test of clinical effectiveness was not the intention of PREVIEW and our preliminary results will feed into deliberations regarding a plausible effect sizes to inform future sample size calculations.

## Conclusion

The PREVIEW pilot and feasibility RCT has produced vital information for the future planning of a robust and successful definitive study. While resuturing was associated with reduced duration to wound healing and improved women's satisfaction with the outcome at 3 months, the size and nature of the study preclude from making reliable estimates of effectiveness. A full trial, informed by PREVIEW feasibility findings, is needed to avoid missing the opportunity to prove the effectiveness of a promising intervention to women.
