# Correction

**DOI:** 10.1136/bmjopen-2016-012766corr1

**Published:** 2017-03-07

Dudley L, Kettle C, Thomas PW*, et al.* Perineal resuturing versus expectant management following vaginal delivery complicated by a dehisced wound (PREVIEW): a pilot and feasibility randomised controlled trial. *BMJ Open *2017;**7:**e012766. doi:10.1136/bmjopen-2016-012766

The superscript “3” should not be next to author Lynn Dudley as this is not one of their affiliations.

