# Supplementary material for: Perineal resuturing versus expectant management following vaginal delivery complicated by a dehisced wound (PREVIEW): a pilot and feasibility randomised controlled trial
Source: BMJ Open. 2017 Feb 10;7(2):e012766. doi: 10.1136/bmjopen-2016-012766 (PMC5306527; doi:10.1136/bmjopen-2016-012766)
Supplement: Supplementary data [file bmjopen-2016-012766supp_data1.pdf]

PREVIEW STUDY

Supplementary data 1: Secondary outcome measures

Additional statistical methods

For dichotomous data, when expected counts for any cell in a table were 5 or more the chi-squared test for association was used to compare resuturing and expectancy groups; otherwise Fisher's Exact test was used.

The aesthetic results of wound healing: a secondary outcome measure

Additional Methods

Women were asked at the pre-specified time points to comment on how they felt about the healing of their perineal wound. The results are presented in tables 1-3 respectively.

Results

Table 1: Women's self-reported assessment of their satisfaction with the aesthetic results of wound healing, (healed or healed poorly) recorded in their 6 week, 3 month and 6 month questionnaires. Comparative data between the two treatment groups (2011-2013).

Satisfaction with the results of wound healing post randomisation: healed or healed poorly	Re-sutured n = 17 n (%)	Expectancy n = 16 n (%)	Odds ratio for poor healing in re-sutured group (95% CI)	*P*-value†
**Perineal healing 6 weeks**			0.10 (0.00,1.96)	0.103F
Felt that perineum had healed	14 (100%)	12 (75%)		
Felt that perineum healed poorly	0 (0%)	4 (25%)		
**Perineal healing 3 months**			0.07 (0.00, 1.44)	0.045F
Felt that perineum had healed	14 (100%)	11 (69%)		
Felt that perineum healed poorly	0 (0%)	5 (31%)		
**Perineal healing 6 months**			0.11 (0.01, 1.44)	0.232F
Felt that perineum had healed	13 (100%)	13 (81%)		
Felt that perineum healed poorly	0 (0%)	3 (19%)		

Re-suturing: self-assessment of perineal healing was completed by 14/17 women at 6 weeks and 3 months (3 women had previously withdrawn) and 13/17 women at 6 months (1 woman did not return her 6 month questionnaire)
†*P*-value F = Fishers exact test

Table 2: Women's self-assessment of their satisfaction with how the wound looked or felt, recorded in their 6 week, 3 month and 6 month questionnaires. Comparative data between the two treatment groups (2011-2013).

Satisfaction with how the wound looked or felt, post randomisation: better or worse	Re-sutured n = 17 n (%)	Expectancy n = 16 n (%)	Odds ratio for worse outcome in re-sutured group (95% CI)	*P*-value†
**6 weeks**			0.20 (0.02, 2.17)	0.322F
Looked or felt better	11 (92%)	9 (69%)		
Looked or felt worse	1 (8%)	4 (31%)		
**3 months**			0.07 (0.00, 1.39)	0.041F
Looked or felt better	11 (100%)	8 (62%)		
Looked or felt worse	0 (0%)	5 (38%)		
**6 months**			0.16 (0.02, 1.66)	0.166F
Looked or felt better	10 (91%)	8 (62%)		
Looked or felt worse	1 (9%)	5 (38%)		

At 6 weeks: not being able to see or feel their perineum was reported by re-suturing n=2 (3 women had also previously withdrawn) and expectancy n=3. At 3 months: not being able to see or feel their perineum was reported by re-suturing n=3 (3 women had previously withdrawn) and expectancy n=2 (variable not completed by n=1 from expectancy). At 6 months: not being able to see or feel their perineum was reported by re-suturing n=2 (3 women had also previously withdrawn and 1 woman did not return her 6 month questionnaire) and expectancy n=2 (variable not completed by n=1 from expectancy).
†*P*-value F = Fishers exact test

Table 3: Women's self-assessment of whether they felt their perineum was back to normal or not, recorded in their questionnaires at 6 week, 3 month and 6 month time points. Comparative data between the two treatment groups (2011-2013).

Did the woman's perineum feel back to normal, post randomisation	Re-sutured n = 17 n (%)	Expectancy n = 16 n (%)	Odds ratio for not back to normal in re-sutured group (95% CI)	*P*-value†
**6 weeks**			0.40 (0.09, 1.83)	0.232C
Yes	10 (71%)	8 (50%)		
No	4 (29%)	8 (50%)		
**3 months**			0.46 (0.10, 2.13)	0.245F
Yes	11 (79%)	8 (53%)		
No	3 (21%)	7 (47%)		
**6 months**			0.18 (0.03, 1.10)	0.114F
Yes	11 (85%)	8 (50%)		
No	2 (15%)	8 (50%)		

Re-suturing: At 6 weeks and 3 months 3 women had previously withdrawn and 1 woman did not return her 6 month questionnaire
†*P*-value C= Chi-square test F = Fishers exact test

Perineal pain: a secondary outcome measure.

Additional methods

Women were asked to report (yes or no) if they were experiencing any perineal pain or discomfort at 2 weeks, 6 weeks, 3 months and 6 months following randomisation.

To increase the validity of recall, at 2 weeks women were asked if they had any perineal pain or discomfort in the previous 24 hours, whilst at the other time points women were asked to report pain or discomfort in the previous week. The comparative results are presented in tables 4 and 5.

Table 4: Women's self-reported assessment of perineal pain following randomisation, recorded in their questionnaires at 2 week, 6 week, 3 month and 6 month time points. Comparative data between the two treatment groups (2011-2013).

Self-reported perineal pain: post randomisation	Re-sutured n=17 n (%)	Expectancy n=16 n (%)	Odds ratio of pain in re-sutured group (95% CI)	*P*-value†
**2 weeks**			0.56 (0.13,2.41)	0.431C
Yes	5 (36%)	8 (50%)		
No	9 (64%)	8 (50%)		
**6 weeks**			1.33 (0.32,5.64)	0.696C
Yes	8 (57%)	8 (50%)		
No	6 (43%)	8 (50%)		
**3 months**			0.40 (0.09,1.83)	0.232C
Yes	4 (29%)	8 (50%)		
No	10 (71%)	8 (50%)		
**6 months**			1.25 (0.07,22.13)	1.000F
Yes	1 (8%)	1 (6%)		
No	12 (92%)	15 (94%)		

Re-suturing: The question was completed by 14/17 women at 6 weeks and 3 months (3/17 women had withdrawn at these time points and 13/17 women at 6 months (1 additional woman did not return her 6 month questionnaire)
†P-value C = Pearson's Chi-square test F = Fishers exact test

Table 5: Women's self-reported assessment of the level of their perineal pain following randomisation, recorded in their questionnaires at 2 week, 6 week, 3 month and 6 month time points. Comparative data between the two treatment groups (2011-2013).

Self-reported level of perineal pain: post randomisation	Re-sutured n=17 n (%)	Expectancy n=16 n (%)
**2 weeks**		
Mild	3 (60%)	5 (63%)
Moderate	2 (40%)	3 (37%)
**6 weeks**		
Mild	7 (57%)	6 (76%)
Moderate	1 (43%)	1 (12%)
Severe	0 (0%)	1 (12%)
**3 months**		
Mild	4 (100%)	7 (88%)
Moderate	0 (0.0%)	1 (12%)
**6 months**		
Mild (*Only includes women who reported pain)*	1 (100%)	1 (100%)

Dyspareunia: a secondary outcome measure

Additional methods

The 6 weeks, 3 months and 6 months mother's questionnaires asked each woman to report if they had resumed sexual intercourse or not. Women who had resumed sexual intercourse were then asked to report if they had any dyspareunia (painful sexual intercourse) either on penetration, deep penetration or around the perineal scar. The comparative rates of resuming sexual intercourse and dyspareunia are presented in table 6.

Results

Table 6: Resuming sexual intercourse and rates of dyspareunia following randomisation, self-reported by women in their 6 week, 3 month and 6 month questionnaires. Comparative data between the two treatment groups who completed the question (2011-2013).

Resuming sexual intercourse (SI) and dyspareunia post randomisation	Re-sutured n=17 n (%)	Expectancy n=16 n (%)	Odds ratio (95% CI)	*P*- value†
**Resumed SI 6 weeks**			1.73 (0.31,9.57)	0.675F
Yes	4 (29%)	3 (19%)		
No	10 (71%)	13 (81%)		
**Dyspareunia 6 weeks**				
Yes	3 (75%)	2 (67%)		
No	1 (25%)	1 (33%)		
**Resumed SI 3 months**			1.67 (0.32,8.74)	0.689F
Yes	11 (79%)	11 (69%)		
No	3 (21%)	5 (31%)		
**Dyspareunia 3 months**				
Yes	6 (55%)	4 (36%)		
No	5 (45%)	7 (64%)		
**Resumed SI 6 months**			Not estimable	
Yes	13 (100%)	16 (100%)		
**Dyspareunia 6 months**				
Yes	11 (85%)	10 (63%)		
No	2 (15%)	6 (37%)		

Re-suturing: The question was completed by 14/17 women at 6 weeks and 3 months (3/17 women had withdrawn at these time points and 13/17 women at 6 months (1 additional woman did not return her 6 month questionnaire.

†*P*-value F = Fishers exact test

Breast feeding rates: a secondary outcome measure

Additional methods

The results in table 7 reveal rates of women who reported breast feeding their baby following delivery and those who were still breast feeding up to 6 months postpartum.

Results

Table 7: Women's self-reported rates of breast feeding recorded in postal questionnaires at 6 weeks, 3 months and 6 months following randomisation. Comparative data between the two treatment groups (2011-2013).

Breast feeding: post randomisation	Re-sutured n = 17 (n %)	Expectancy n = 16 (n %)	Odds ratio (95% CI)	*P*- value†
**Breast fed since delivery**			7.00 (1.14, 42.97)	0.046
Yes	7 (50%)	14 (88%)		
No	7 (50%)	2 (12%)		
**Breast feeding at 6 weeks** *(if commenced at delivery*			1.39 (0.19, 9.97)	1.000
Yes	5 (71%)	9 (64%)		
No	2 (29%)	5 (36%)		
**Breast feeding at 3 months** *(if commenced at delivery)*			1.00 (0.16, 6.25)	1.000
Yes	4 (57%)	8 (57%)		
No	3 (43%)	6 (43%)		
**Breast feeding at 6 months** *(if commenced at delivery*			1.33 (0.21, 8.29)	1.000
Yes	4 (57%)	7 (50%)		
No	3 (43%)	7 (50%)		

Re-suturing: The question was completed by 14/17 women at 6 weeks and 3 months (3/17 women had withdrawn at these time points and 13/17 women at 6 months (1 additional woman did not return her 6 month questionnaire.
P-value F = Fishers exact test
