# Supplementary material for: Perineal resuturing versus expectant management following vaginal delivery complicated by a dehisced wound (PREVIEW): a pilot and feasibility randomised controlled trial
Source: BMJ Open. 2017 Feb 10;7(2):e012766. doi: 10.1136/bmjopen-2016-012766 (PMC5306527; doi:10.1136/bmjopen-2016-012766)
Supplement: Supplementary data [file bmjopen-2016-012766supp_data2.pdf]

PREVIEW STUDY

Supplementary data 2: Antibiotics prescribed

Type of antibiotics prescribed per trial group for women with dehisced perineal wounds at or prior to randomisation (2011-2013).

Re-sutured: type of antibiotics prescribed	Number of women prescribed antibiotic
Co-amoxiclav	6
Flucloxacillin	1
Cephalexin and metronidazole	1
Erythromycin	1
Metronidazole	1
Metronidazole and Co-amoxiclav	1
Metronidazole and Erythromycin	1

Expectancy: type of antibiotics prescribed	Number of women prescribed antibiotic
Co-amoxiclav	5
Flucloxacillin	2
Cephalexin and metronidazole	1
Cephalexin	2
Erythromycin	1
Information not available	3
